# PyNeval: A Python Toolbox for Evaluating Neuron Reconstruction Performance

**DOI:** 10.3389/fninf.2021.767936

**Published:** 2022-01-28

**Authors:** Han Zhang, Chao Liu, Yifei Yu, Jianhua Dai, Ting Zhao, Nenggan Zheng

**Affiliations:** ^1^Qiushiq Academy for Advanced Studies (QAAS), Zhejiang University, Hangzhou, China; ^2^College of Computer Science and Technology, Zhejiang University, Hangzhou, China; ^3^Zhejiang Lab, Hangzhou, China; ^4^Collaborative Innovation Center for Artificial Intelligence by MOE and Zhejiang Provincial Government (ZJU), Hangzhou, China; ^5^Howard Hughes Medical Institute, Janelia Research Campus, Ashburn, VA, United States

**Keywords:** PyNeval, metric, quantitative analysis, neuron tracing, neuron reconstruction, toolbox

## Abstract

Quality assessment of tree-like structures obtained from a neuron reconstruction algorithm is necessary for evaluating the performance of the algorithm. The lack of user-friendly software for calculating common metrics motivated us to develop a Python toolbox called PyNeval, which is the first open-source toolbox designed to evaluate reconstruction results conveniently as far as we know. The toolbox supports popular metrics in two major categories, geometrical metrics and topological metrics, with an easy way to configure custom parameters for each metric. We tested the toolbox on both synthetic data and real data to show its reliability and robustness. As a demonstration of the toolbox in real applications, we used the toolbox to improve the performance of a tracing algorithm successfully by integrating it into an optimization procedure.

## 1. Introduction

Reconstructing tree structures of labeled neurons in light microscope images is a critical step for neuroscientists to study neural circuits (Parekh and Ascoli, 2013; Peng et al., 2015). Researchers have longed for automating this process of neuron reconstruction, also called neuron tracing, to overcome the bottleneck of manual annotation or proofreading (Gillette et al., 2011b; Peng et al., 2011). Despite decades of efforts (Halavi et al., 2012; Acciai et al., 2016), however, there is still no computer algorithm that can be as reliable as human labor. Besides being a complex computer vision problem itself, neuron tracing has baffled developers on how an algorithm should be evaluated. Unlike many image segmentation problems, neuron tracing has no universally accepted metric to measure its performance. In fact, it is infeasible to design one metric for all applications, which have different tolerance to different types of reconstruction errors. The real problem here is a lack of easy access to evaluation metrics. As a result, researchers have to implement a metric by themselves or compromise on metric properness for convenience. This has caused two issues in the literature. First, performance evaluation was often limited to one or two metrics that were not sufficient to offer comprehensive comparisons. Second, the metrics applied were ambiguous in general without open implementations, causing potential inconsistency and low reproducibility.

This problem can be addressed by open-source user-friendly software that allows evaluating neuron reconstruction qualities in various ways. Such software should cover the two major categories of reconstruction metrics, geometrical metrics and topological metrics. Geometrical metrics measure how well a reconstructed model overlaps with the underlying gold standard or ground truth model, while topological metrics measure the topological similarity between the two models. Geometrical metrics are often computed by summarizing spatial matching between the two models, such as counting the number of matched nodes as done in the popular substantial spatial distance (SSD) metric (Peng et al., 2010) or measuring the length of overlapped branches in the so called length metric (Wang et al., 2011). These metrics are straightforward for telling where branches are missing or over-traced in reconstruction, but they are not suitable for evaluating topological accuracy, which is crucial in some applications such as electrophysiological simulation. For the latter situation, topological metrics such as the Digital Reconstruction of Axonal and Dendritic Morphology (DIADEM) metric (Gillette et al., 2011a), tree edit distance (Bille, 2005), and critical node (CN) metric (Feng et al., 2015) are preferred.

Hence, we introduce a Python toolbox called PyNeval, which is the first open-source toolbox designed to provide multiple choices for evaluating the qualities of reconstruction results conveniently. In specific, PyNeval is designed to have the following features:

- PyNeval has a user-friendly command-line interface for easy use and a flexible way of configuring parameters for covering a broad range of user requirements.
- PyNeval provides various evaluation methods for measuring both geometrical and topological qualities of reconstructions.
- PyNeval provides an interface for optimizing any reconstruction algorithm that converts an image into an SWC file with adjustable parameters.

In this paper, we formulate each evaluation method implemented in PyNeval under a mathematical framework if it has not been clearly defined in the literature. Our implementation follows those formulations, which give users a clear and unambiguous picture of what PyNeval computes. We apply PyNeval to randomly perturbed data to show that PyNeval can produce reliable evaluation scores from different metrics. The difference among the metrics can be seen in their results of manually-designed special cases. Besides comparing different tracing algorithms, PyNeval can be used to optimize any reconstruction algorithm with tunable parameters, as demonstrated in our experiment on mouse brain data acquired by fMOST (Gong et al., 2016).

## 2. Method

### 2.1. SWC Format

The PyNeval toolbox is designed based on the SWC format (Cannon et al., 1998), the common format of neuron reconstruction results. The format represents the shape of a neuron in a tree structure that consists of a set of hierarchically organized nodes (Feng et al., 2015):


(1) $$\begin{array}{l} T=\{n_{i}=(x_{i},r_{i},n_{j})|i=1,...,N_{T},n_{j}\in T\cup n_{0},i\neq j,\\ x_{i}\in {\mathbb{R}}^{3},r_{i}\in \mathbb{R}\} \end{array}$$

where *N*_*T*_ = |*T*| is the number of nodes of *T*, the *i*th node **n**_*i*_ is a sphere centering at **x**_*i*_ = (*x*_*i*_, *y*_*i*_, *z*_*i*_) with radius *r*_*i*_, and **n**_0_ is a virtual node. In this definition, **n**_*j*_ is called the parent of **n**_*i*_, and a node with a virtual node as its parent is called a root node. For convenience, we also define the following functions:

- Parent of a node: ρ:(**x**_*i*_, *r*_*i*_, **n**_*j*_) ∈ *T* ↦ **n**_*j*_ ∈ *T* ∪ **n**_0_
- Position of a node: $\text{x}:\left({\text{x}}_{i},r_{i},{\text{n}}_{j}\right)\in T\mapsto {\text{x}}_{i}\in {\mathbb{R}}^{3}$
- Radius of a node: *r*:(**x**_*i*_, *r*_*i*_, **n**_*j*_) ∈ *T* ↦ *r*_*i*_ ∈ ℝ

The edge set of the model *T* is defined as


(2) $$\begin{array}{l} E\left(T\right)=\left\{{\text{e}}_{i}|{\text{e}}_{i}=\left({\text{n}}_{i},\rho \left({\text{n}}_{i}\right)\right),{\text{n}}_{i}\in T\right\} \end{array}$$

One important constraint on the SWC model *T* is that the graph *G* = (*T* ∪ **n**_**0**_, *E*(*T*)) has no loop, which means that it is a tree.

### 2.2. Software Design

**Figure 1:**
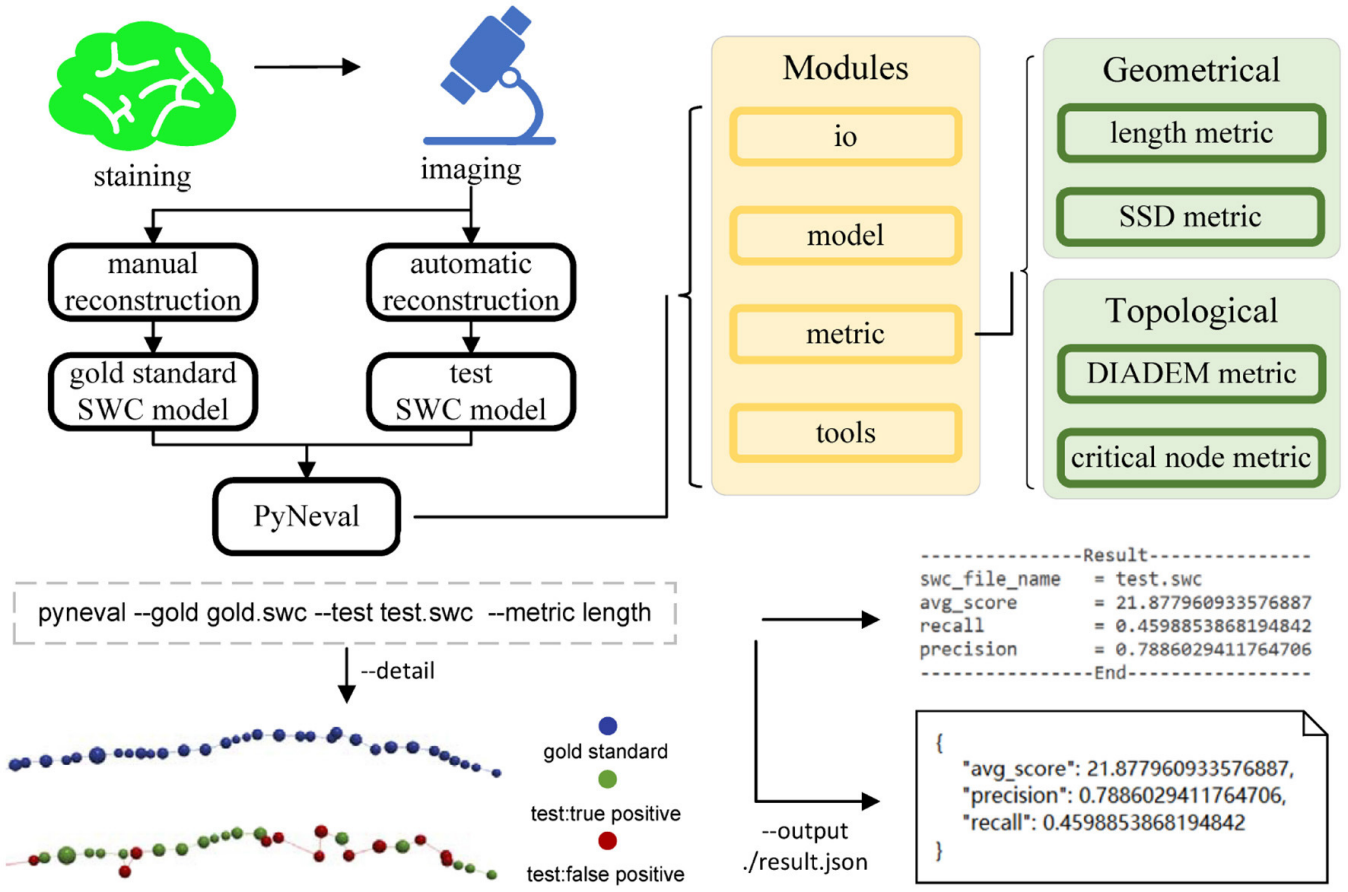
The overview of PyNeval, which can take a gold standard model and a test model from the same image as input through a command line interface and outputs quality scores of the test model, as well as more details about reconstruction errors. Four different metrics, including the length and substantial spatial distance (SSD) metrics in the geometrical category and the DIADEM and critical node (CN) metrics in the topological category, are available in PyNeval.

Assuming that the reconstruction results are in the SWC format, PyNeval takes a gold standard SWC file as well as one or more testing SWC files and outputs the quality scores for each testing SWC. Since PyNeval supports multiple metrics, it should also allow the user to specify metric options. As a consequence, input SWC files and metric options form the essential parameters of the main PyNeval command. While this provides a straightforward interface for an application, it is not flexible enough to adapt to more subtle user requirements such as setting specific parameters for a certain metric or checking evaluation details. Therefore, PyNeval has a flexible but friendly way of accepting optional parameters, allowing the user to specify these parameters without having to check extensive documents. PyNeval can output carefully formatted results to the screen for easy reading or save the results with more details to a file for further analysis, depending on the user's choice of the output parameters. For example, the –detail option can be used to produce an SWC file that labels each node in the test structure with a specific type to indicate what kind of error is associated with that node. The overall architecture of PyNeval is shown in Figure 1.

### 2.3. Metrics

PyNeval supports four commonly used metrics in both geometrical and topological categories, although it can be easily extended to more metrics. To explain the metrics implemented in PyNeval unambiguously, we use the notations listed in Table 1.

**Table 1:** Mathematical notations used for explaining the metrics.

**Symbol**	**Meaning**
*T*_*g*_	Gold standard SWC model
*T*_*t*_	SWC model for evaluation
**n**_*i*_	A node in a SWC model with an unique index *i*
*E*(*T*)	Set of all edges in *T*
**e**_*i*_	Edge from node **n**_*i*_ to node ρ(**n**_*i*_)
*d*(*x, y*)	Distance between two objects, which can be nodes, edges or trees
*L*	Length of an edge or an edge set
*M*	Matched node or edge set
I	Interpolation function

More specifically, some of the notations can be interpreted as follows:

- Besides always assuming that **e**_*ij*_ = (**n**_*i*_, **n**_*j*_) and **e**_*i*_ = (**n**_*i*_, ρ(**n**_*i*_)), we also use **n** and **e** to represent a node and an edge, respectively, when there is no need to index them.
- Node interpolation
  (3) $$\begin{array}{l} I(n,\lambda )=\{\begin{array}{ll} \left((1-\lambda )x(n\right) & +\lambda x(\rho (n)),(1-\lambda )r(n)\\ & +\lambda r(\rho (n)),\rho (n))),0\leq \lambda <1\\ \rho (n), & \lambda =1 \end{array} \end{array}$$
- Interpolation between two nodes, no matter if they are connected
  (4) $$\begin{array}{l} I(n_{i},n_{j},\lambda )=\{\begin{array}{lll} ((1-\lambda )x_{i} & +\lambda x_{j},(1-\lambda )r_{i} & \\ & +\lambda r_{j},n_{j}), & 0\leq \lambda <1\\ n_{j}, & & \lambda =1 \end{array} \end{array}$$
- Node distances
  (5) $$\begin{array}{l} d\left({\text{n}}_{i},{\text{n}}_{j}\right)=\sqrt{{\left(x_{i}-x_{j}\right)}^{2}+{\left(y_{i}-y_{j}\right)}^{2}+{\left(z_{i}-z_{j}\right)}^{2}} \end{array}$$
  (6) $$\begin{array}{l} d_{xy}\left({\text{n}}_{i},{\text{n}}_{j}\right)=\sqrt{{\left(x_{i}-x_{j}\right)}^{2}+{\left(y_{i}-y_{j}\right)}^{2}} \end{array}$$
  (7) $$\begin{array}{l} d_{z}\left({\text{n}}_{i},{\text{n}}_{j}\right)=|z_{i}-z_{j}| \end{array}$$
  (8) $$\begin{array}{l} d\left(\text{n},{\text{e}}_{i}\right)=\underset{\lambda }{\min}d\left(\text{n},I\left({\text{n}}_{i},\lambda \right)\right) \end{array}$$
  (9) $$\begin{array}{l} d\left(\text{n},T\right)=\underset{\text{e}\in E\left(T\right)}{\min}d\left(\text{n},\text{e}\right) \end{array}$$
- Node length
  (10) $$\begin{array}{l} L\left(\text{n}\right)=\{\begin{array}{cc} 0, & \text{n}\text{ is a root node}\\ d\left(\text{n},\rho \left(\text{n}\right)\right), & \text{otherwise} \end{array} \end{array}$$
- Edge lengths
  (11) $$\begin{array}{l} L\left({\text{e}}_{ij}\right)=d\left({\text{n}}_{i},{\text{n}}_{j}\right) \end{array}$$
  (12) $$\begin{array}{l} L\left(E\left(T\right)\right)=\underset{\text{e}\in E\left(T\right)}{\sum }L\left(\text{e}\right) \end{array}$$
- Tree length
  (13) $$\begin{array}{l} L\left(T\right)=L\left(E\left(T\right)\right) \end{array}$$

#### 2.3.1. Length Metric

It is natural to evaluate the quality of a reconstruction *T*_*t*_ by measuring how well its branches overlap with the gold standard model *T*_*g*_. This can be computed by matching edges between *T*_*t*_ and *T*_*g*_ and then summing up the lengths of the matched edges in *T*_*t*_ and *T*_*g*_, respectively, to produce the common precision and recall metrics. Before proceeding to explain the length metric in detail, we first need to define some more notations

- A segment lying on an edge (**n**, ρ(**n**)) is
  (14) $$\begin{array}{l} C\left(\text{n},\lambda_{1},\lambda_{2}\right)=\left\{\text{x}\left(I\left(\text{n},\lambda \right)\right)|0\leq \lambda_{1}\leq \lambda \leq \lambda_{2}\leq 1\right\} \end{array}$$
  and its length is *L*(*C*(**n**, λ_1_, λ_2_)) = (λ_2_−λ_1_)*L*(**n**).
- The overlap ratio between two segments *C*_1_, *C*_2_ with respect to the edge (**n**, ρ(**n**)) is defined as $O\left(C_{1},C_{2}\right)$. Suppose that *C*_1_ = (**n**_*i*_, α_1_, α_2_), *C*_2_ = *C*(**n**_*j*_, β_1_, β_2_), the overlap ratio $O\left(C_{1},C_{2}\right)$ is
  (15) $$\begin{array}{l} O(C_{1},C_{2})=\{\begin{array}{ll} \max(0,\min(\alpha_{2}-\alpha_{1}, & \\ \beta_{2}-\beta_{1},\alpha_{2}-\beta_{1},\beta_{2}-\alpha_{1})), & i=j\\ 0, & i\neq j \end{array} \end{array}$$
- A simple path between two points on a tree is
  (16) $$\begin{array}{l} P\left(\left({\text{n}}_{s},\lambda_{s}\right),\left({\text{n}}_{t},\lambda_{t}\right)\right)=\{\begin{array}{cc} \left\{C\left({\text{n}}_{s},\min\left(\lambda_{s},\lambda_{t}\right),\max\left(\lambda_{s},\lambda_{t}\right)\right)\right\}, & s=t\\ \left\{C\left({\text{n}}_{i_{k}},\alpha_{k},\beta_{k}\right)|k=1\cdots K\right\}, & s\neq t \end{array} \end{array}$$
  where *i*_1_ = *s*, *i*_*K*_ = *t*, **n**_*i*_*k*−1__ = ρ(**n**_*i*_*k*__)or**n**_*i*_*k*__ = ρ(**n**_*i*_*k*−1__), *K* is the number of edges on the path and
  (17) $$\begin{array}{l} \left(\alpha_{k},\beta_{k}\right)=\{\begin{array}{cc} \left(0,\lambda_{s}\right), & k=1\text{ and }{\text{n}}_{s}=\rho \left({\text{n}}_{i_{2}}\right)\\ \left(\lambda_{s},1\right), & k=1\text{ and }\rho \left({\text{n}}_{s}\right)={\text{n}}_{i_{2}}\\ \left(0,\lambda_{t}\right), & k=K\text{ and }{\text{n}}_{t}=\rho \left({\text{n}}_{i_{K-1}}\right)\\ \left(\lambda_{t},1\right), & k=K\text{ and }\rho \left({\text{n}}_{t}\right)={\text{n}}_{i_{K-1}}\\ \left(0,1\right), & \text{otherwise} \end{array} \end{array}$$

In our implementation, we construct the matched edge set between *T*_*t*_ and *T*_*g*_ as demonstrated in Algorithm 1.

**Algorithm 1:** Length metric.

**Input:** $T_{g},T_{t},\epsilon_{l}\in {\mathbb{R}}^{+},\epsilon_{o}\in {\mathbb{R}}^{+},\epsilon_{d}\in {\mathbb{R}}^{+}$
**Output:** precision, recall
1: *M*_*t*_ ← ∅, *M*_*g*_ ← ∅
2: **for** **e**_*ij*_ in *E*(*T*_*t*_) **do**
3: $I\left({\text{n}}_{g_{1}}^{\prime },\lambda_{1}\right)\leftarrow {\text{arg min}}_{{\text{n}}^{\prime }\in \cup_{\text{n}\in T_{g}}\left\{I\left(\text{n},\lambda \right)|0\leq \lambda \leq 1\right\}}d\left({\text{n}}_{i},{\text{n}}^{\prime }\right)$
4: $I\left({\text{n}}_{g_{2}}^{\prime },\lambda_{2}\right)\leftarrow {\text{arg min}}_{{\text{n}}^{\prime }\in \cup_{\text{n}\in T_{g}}\left\{I\left(\text{n},\lambda \right)|0\leq \lambda \leq 1\right\}}d\left({\text{n}}_{j},{\text{n}}^{\prime }\right)$
5: **if** $\max\left(d\left({\text{n}}_{i},I\left({\text{n}}_{g_{1}}^{\prime },\lambda_{1}\right)\right),d\left({\text{n}}_{j},I\left({\text{n}}_{g_{2}}^{\prime },\lambda_{2}\right)\right)\right)<\epsilon_{d}$ **then**
6: $P\left(\left({\text{n}}_{g_{1}}^{\prime },\lambda_{1}\right),\left({\text{n}}_{g_{2}}^{\prime },\lambda_{2}\right)\right)$ is the simple path between $I\left({\text{n}}_{g_{1}}^{\prime },\lambda_{1}\right)$ and $I\left({\text{n}}_{g_{2}}^{\prime },\lambda_{2}\right)$
7: **if** $\frac{\left|L(e_{ij})-L(P((n_{g_{1}}^{\prime },\lambda_{1}),(n_{g_{2}}^{\prime },\lambda_{2}))\right|}{L(e_{ij})}<\varepsilon_{l}$ and $\underset{C_{1}\in P\left(\left({\text{n}}_{g_{1}}^{\prime },\lambda_{1}\right),\left({\text{n}}_{g_{2}}^{\prime },\lambda_{2}\right)\right),C_{2}\in M_{g}}{\max}O\left(C_{1},C_{2}\right)<\varepsilon_{o}$ **then**
8: *M*_*t*_ ← *M*_*t*_ ∪ {**e**_*ij*_}
9: $M_{g}\leftarrow M_{g}\cup P\left(\left({\text{n}}_{g_{1}}^{\prime },\lambda_{1}\right),\left({\text{n}}_{g_{2}}^{\prime },\lambda_{2}\right)\right)$
10: **end if**
11: **end if**
12: **end for**
13: $\text{precision}\leftarrow \frac{L\left(M_{t}\right)}{L\left(T_{t}\right)}$
14: $\text{recall}\leftarrow \frac{L\left(M_{g}\right)}{L\left(T_{g}\right)}$
15: **return** precision, recall

#### 2.3.2. SSD Metric

The SSD metric (Peng et al., 2010) can be viewed as a variant of the length metric in terms of what it tries to measure. Instead of matching edges directly, however, SSD counts how many nodes are matched without excluding duplicated matches. Besides, SSD provides an additional metric to measure how far the unmatched nodes are away from the counterpart model. One extra step of SSD metric is resampling each branch of *T*_*t*_ and *T*_*g*_ uniformly to reach a sufficient density ε_*sp*_


(18) $$\begin{array}{l} R\left(T\right)=\left\{{\text{n}}_{k}^{\left(i\right)}|{\text{n}}_{i}\in T,k=0,1,\ldots ,K_{i}\right\} \end{array}$$

where ${\text{n}}_{k}^{\left(i\right)}=I\left({\text{n}}_{i},{\text{n}}_{k+1}^{\left(i\right)},\frac{k}{k+1}\right)$, ${\text{n}}_{K_{i}}^{\left(i\right)}={\text{n}}_{0}^{\left(j\right)}$, ρ(**n**_*i*_) = **n**_*j*_, *K*_*i*_ ε _*sp*_ ≤ *L*(**e**_*ij*_), and (*K*_*i*_ + 1) ε _*sp*_ > *L*(**e**_*ij*_).

After that, like computing the length metric, the SSD metric can be obtained by constructing the matched node set *M*_**n**_ between two SWC models *T*_*g*_ and *T*_*t*_ shown in Algorithm 2.

**Algorithm 2:** SSD metric.

**Input:** $R\left(T_{g}\right),R\left(T_{t}\right),\epsilon_{sp}\in {\mathbb{R}}^{+},\epsilon_{ssd}\in {\mathbb{R}}^{+}$
**Output:** precision, recall, SSD cost
1: *M*_*n*_(*T*_*g*_, *T*_*t*_)←∅, *M*_*n*_(*T*_*t*_, *T*_*g*_)←∅
2: **for** **n**_*i*_ in $R\left(T_{g}\right)$ **do**
3: **if** $\underset{{\text{n}}_{j}\in R\left(T_{t}\right)}{\min}d\left({\text{n}}_{i},{\text{n}}_{j}\right)<\varepsilon_{ssd}$ **then**
4: *M*_*n*_(*T*_*g*_, *T*_*t*_) ← *M*_*n*_(*T*_*g*_, *T*_*t*_) ∪ {**n**_*i*_}
5: **end if**
6: **end for**
7:
8: **for** **n**_*i*_ in $R\left(T_{t}\right)$ **do**
9: **if** $\underset{{\text{n}}_{j}\in R\left(T_{g}\right)}{\min}d\left({\text{n}}_{i},{\text{n}}_{j}\right)<\varepsilon_{ssd}$ **then**
10: *M*_*n*_(*T*_*t*_, *T*_*g*_) ← *M*_*n*_(*T*_*t*_, *T*_*g*_) ∪ {**n**_*i*_}
11: **end if**
12: **end for**
13:
14: $\text{precision}\leftarrow \frac{\left|M_{n}\left(R\left(T_{t}\right),R\left(T_{g}\right)\right)\right|}{\left|R\left(T_{t}\right)\right|}$
15: $\text{recall}\leftarrow \frac{\left|M_{n}\left(R\left(T_{g}\right),R\left(T_{t}\right)\right)\right|}{\left|R\left(T_{g}\right)\right|}$
16: $\text{SSD cost}\leftarrow \frac{SSD\left(R\left(T_{t}\right),R\left(T_{g}\right)\right)+SSD\left(R\left(T_{g}\right),R\left(T_{t}\right)\right)}{2}$
17: **return** precision, recall, SSD cost

#### 2.3.3. CN Metric

The CN metric measures how many CNs are reconstructed correctly. A critical node is either a branching or terminal node, which determines the topology of an SWC model. Mathematically, the set of the CNs of an SWC model *T* is defined as


(19) $$\begin{array}{l} K\left(T\right)=\left\{\text{n}|\text{n}\in T,D_{T}\left(\text{n}\right)\neq 2\right\} \end{array}$$

where *D*_*T*_(**n**) is the degree of node **n** in the tree *T*.

With the CNs, we can compute the CN metric with Algorithm 3.

**Algorithm 3:** Critical node metric.

**Input:** $K\left(T_{g}\right),K\left(T_{t}\right),\epsilon_{br}\in {\mathbb{R}}^{+}$
**Output:** precision, recall
1: $V_{b}\leftarrow K\left(T_{t}\right)\cup K\left(T_{g}\right)$
2: $E_{b}\leftarrow \left\{\left({\text{n}}^{\left(t\right)},{\text{n}}^{\left(g\right)}\right)|{\text{n}}^{\left(t\right)}\in K\left(T_{t}\right),{\text{n}}^{\left(g\right)}\in K\left(T_{g}\right),d\left({\text{n}}^{\left(t\right)},{\text{n}}^{\left(g\right)}\right)<\varepsilon_{br}\right\}$
3: *G*_*b*_←(*V*_*b*_, *E*_*b*_)
4: $M_{b}^{*}\leftarrow {\text{arg max}}_{M_{b}}|M_{b}|$ **#***M*_*b*_ is a matching in *G*_*b*_, i.e., *M*_*b*_ is a subgraph of *G*_*b*_ and all of its nodes
5: have degree 1.
6: $\text{precision}\leftarrow \frac{\left|M_{b}^{*}\right|}{\left|K\left(T_{t}\right)\right|}$
7: $\text{recall}\leftarrow \frac{\left|M_{b}^{*}\right|}{\left|K\left(T_{g}\right)\right|}$
8: **return** precision, recall

#### 2.3.4. DIADEM Metric

Introduced by Gillette et al. (2011a) for the DIADEM challenge (Gillette et al., 2011b), the DIADEM metric evaluates the similarity between two models by comparing their branching structures. Like the CN metric, the DIADEM metric is also based on matching CNs in $K\left(T_{g}\right)$ and $K\left(T_{t}\right)$, here K(T) is defined in equation (19). But its matching criteria are more complicated than simply checking the distances. A brief description of the DIADEM metric is proposed as Algorithm 4.

**Algorithm 4:** Diadem metric.

**Input:** $K\left(T_{g}\right),K\left(T_{t}\right),\epsilon_{xy}\in {\mathbb{R}}^{+},\epsilon_{z}\in {\mathbb{R}}^{+},,\epsilon_{ld}\in {\mathbb{R}}^{+}$
**Output:** DIADEM score
1: **for** ${\text{n}}_{i}\in K\left(T_{g}\right)$ **do**
2: **for** ${\text{n}}_{j}\in K\left(T_{t}\right)$ **do**
3: **if** *d*_*xy*_(**n**_*i*_, **n**_*j*_) < ϵ_*xy*_ and *d*_*z*_(**n**_*i*_, **n**_*j*_) < ϵ_*z*_ **then**
4: **#** search for α(**n**), the ancestor of **n** on the path between **n** and its root **n**_0_.
5: **#** ${\text{n}}_{0}^{\left(g\right)}$, ${\text{n}}_{0}^{\left(t\right)}$ are the roots of gold and test trees respectively.
6: **for** α(**n**_*i*_) in $P\left(\left({\text{n}}_{i},0\right),\left({\text{n}}_{0}^{\left(g\right)},0\right)\right)$ **do**
7: **for** α(**n**_*j*_) in $P\left(\left({\text{n}}_{j},0\right),\left({\text{n}}_{0}^{\left(t\right)},0\right)\right)$ **do**
8: **if** α(**n**_*i*_) matches α(**n**_*j*_) and $\frac{\left|L\left(P\left({\text{n}}_{i},\alpha \left({\text{n}}_{i}\right)\right)\right)-L\left(P\left({\text{n}}_{j},\alpha \left({\text{n}}_{j}\right)\right)\right)\right|}{L\left(P\left({\text{n}}_{i},\alpha \left({\text{n}}_{i}\right)\right)\right)}<\epsilon_{ld}$ **then**
9: *M*_*d*_ ← *M*_*d*_ ∪ {**n**_*i*_}
10: **end if**
11: **end for**
12: **end for**
13: **end if**
14: **end for**
15: **end for**
16: $\text{DIADEM score}=\frac{\underset{\text{n}\in M_{d}}{\sum }D_{T_{g}}\left(\text{n}\right)}{\underset{\text{n}\in K\left(T_{g}\right)}{\sum }D_{T_{g}}\left(\text{n}\right)}$
17: **return** DIADEM score

There are also several rounds of scanning to deal with the problem that ${\text{n}}_{j}\in K\left(T_{t}\right)$ is not the only node that meets the conditions, and labels every unmatched CN in *T*_*g*_ as a match if it is on a matched path. More details can be found in the reference (Gillette et al., 2011a).

### 2.4. Implementation

PyNeval is implemented in Python 3 (Oliphant, 2007) using several powerful open-source packages, including Numpy (Van Der Walt et al., 2011) for numerical computation, Anytree (Anytree., 2020) for handling the SWC data structure, and kdtree as well as Rtree (KDtree., 2017; Rtree., 2020) for fast search of closest edges and nodes.

## 3. Results

### 3.1. Robustness Test

We applied PyNeval to randomly perturbed gold standard reconstructions to characterize each metric and evaluate the robustness of our program. The perturbed dataset is constructed by randomly moving a portion of nodes in the original reconstructions, which are gold standard SWC models from the standard BigNeuron dataset (Peng et al., 2015) as well as our custom dataset acquired from fMOST (Gong et al., 2016). As listed in Table 2, a total of six reconstructions with a large variety of sizes were used for the test.

**Table 2:** Summary of neuron reconstructions from six image stacks.

**ID**	**Number of nodes**	**Number of roots**	**Source**
BN1	4,966	7	BigNeuron
BN2	852	7	BigNeuron
BN3	432	2	BigNeuron
BN4	4,251	4	BigNeuron
FM1	5,160	63	fMOST
FM2	674	9	fMOST

**Figure 2:**
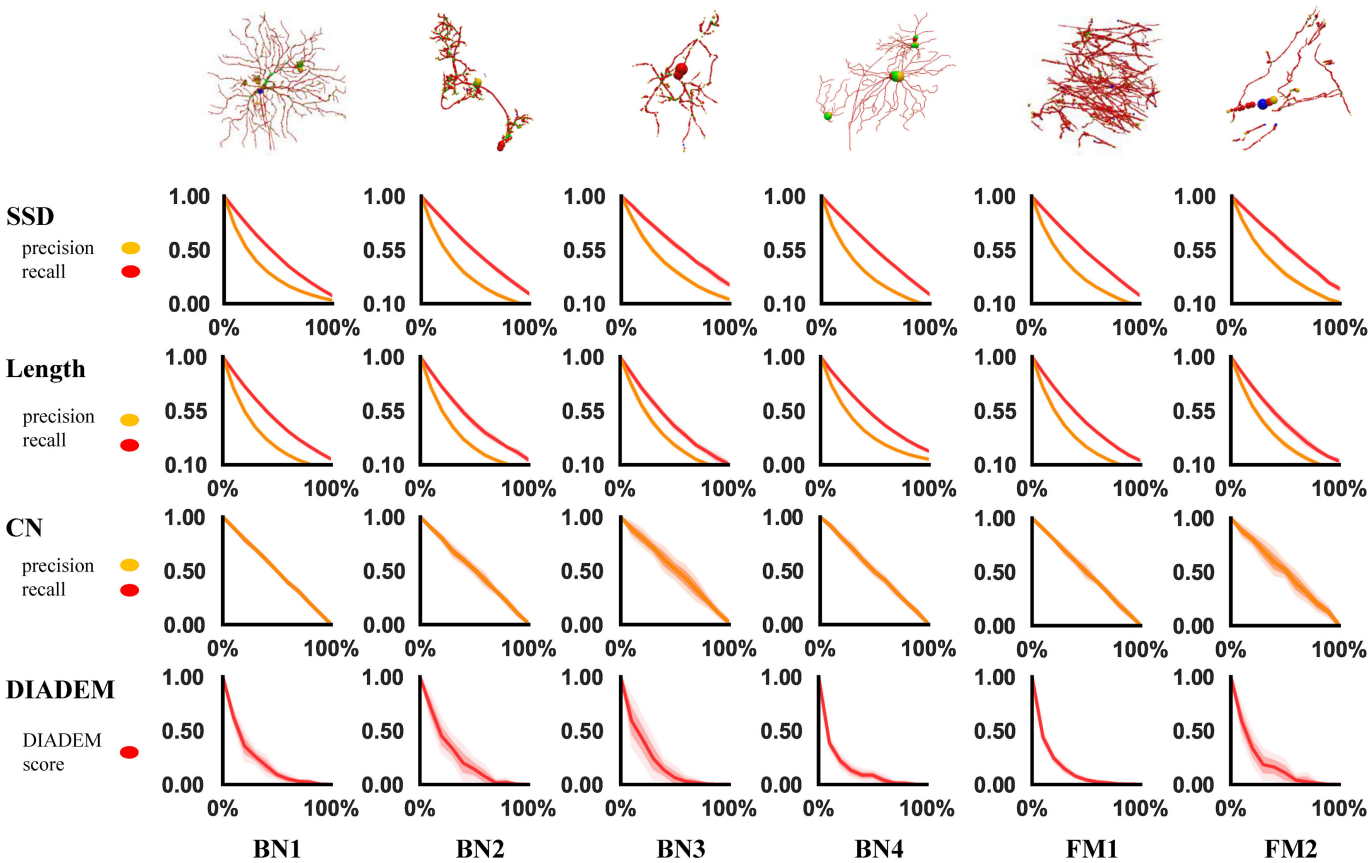
Result of robustness test. Each row represents a metric and each column represents an swc model. In each chart, the x-axis is the perturbed proportion and the y-axis is the corresponding metric value.

A reasonable metric should produce decreasing quality scores as the perturbation ratio increases. This can be seen in the experimental results plotted in Figure 2, in which each curve shows the trend of a metric score along the increasing perturbation ratio. Each metric score at a perturbation ratio was averaged from 10 trials for a sequence of 11 perturbation ratios increasing by the step of 0.1 from 0 to 1. As expected, the curves are consistently similar among different models, in spite of their different morphologies. They all follow the right trend that more perturbation results in a worse score. We can also see that, topological metrics have higher variance than geometrical metrics, which is not surprising because how a perturbation affects the topology highly depends on the positions of the perturbed nodes. This suggests that when we use a topological metric to evaluate an algorithm, more samples or trials might be needed to draw a reliable conclusion.

### 3.2. Special Case Analysis

**Figure 3:**
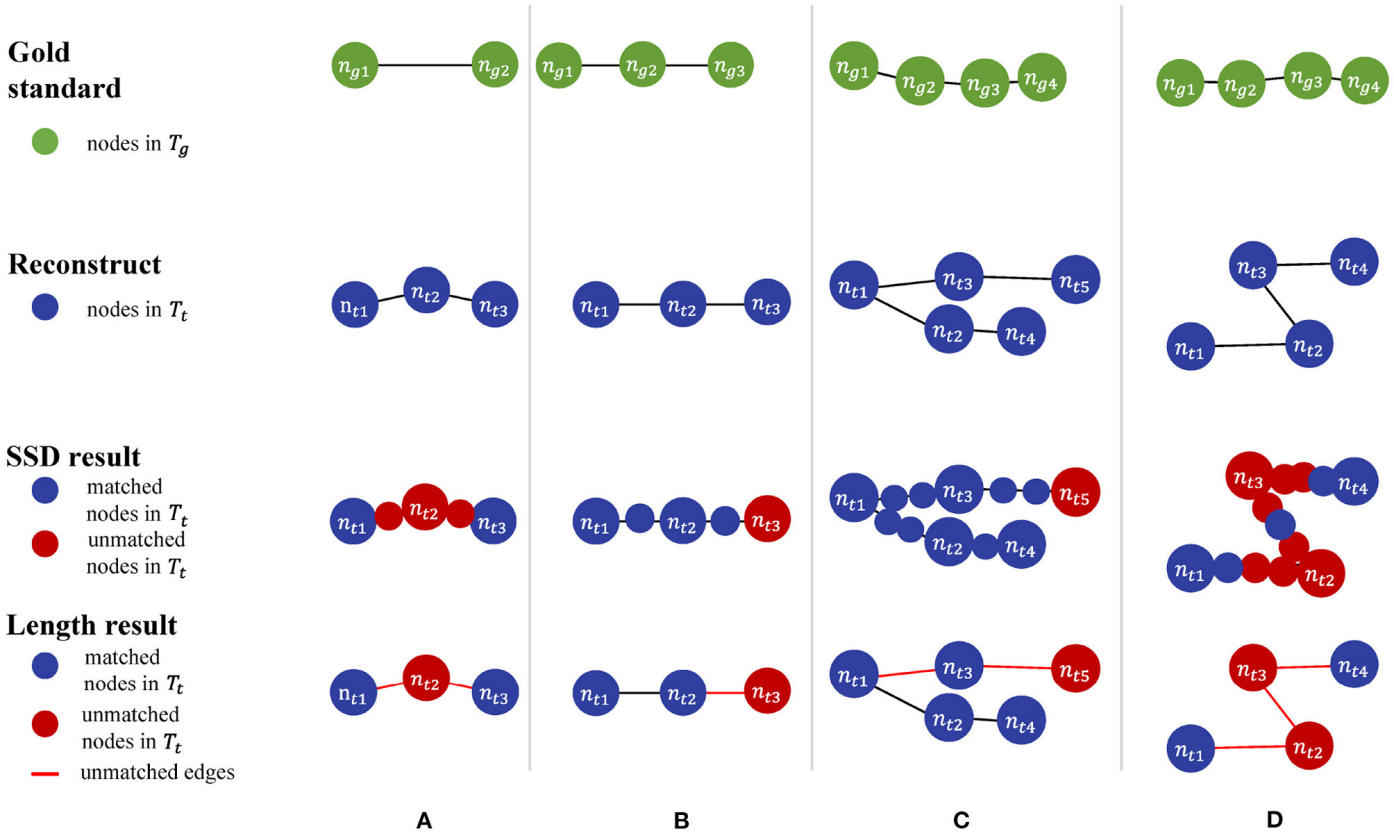
**(A–D)** Four manually constructed cases for testing geometrical metrics.

In addition to the perturbation experiment, we also tested the behaviors of the metrics on some special cases to show their differences more clearly. We constructed four special cases for geometrical metrics and the other four for topological metrics, including Figure 3:

- Test cases for geometrical metrics
  1. Both ends of an edge in *T*_*g*_ have matched nodes in *T*_*t*_, but *T*_*t*_ has an extra node that deviates the path from the edge segment in *T*_*g*_.
  2. *T*_*g*_ manages to find a match path in *T*_*t*_, but its nodes do not match those on the same path in *T*_*g*_ due to the sampling rate.
  3. A straight path in *T*_*g*_ is reconstructed as a bifurcation in *T*_*t*_ by mistake.
  4. *T*_*t*_ distorts a relatively straight path in *T*_*g*_ into a zigzag path.
- Test cases for topological metrics
  1. The nodes are matched but a wrong connection in *T*_*t*_ changes the root to a non-CN.
  2. *T*_*t*_ has wrong connections, but all the CNs are still matched between *T*_*t*_ and *T*_*g*_.
  3. The reconstruction moves a node and all its descendants to a different location.
  4. Connection mistakes in *T*_*t*_ break the original model into several isolated graphs.

Table 3 shows different results on the same special cases produced by the SSD and length metrics. The SSD metric tends to output higher F1 scores than the length metric does, but it is not necessarily better or worse. In some cases (Figures 3A,B), the SSD scores look more reasonable because their more granulated matching can capture partial matching of a path. In other cases (Figures 3C,D), where the errors are more complicated, however, the SSD metric can overestimate reconstruction qualities by counting duplicated matches.

**Table 3:** PyNeval results of the SSD and length metrics for the geometrical cases are shown in Figures 3A–D.

**Method**	**Index**	**File name**			
		**A**	**B**	**C**	**D**
SSD metric	SSD score	1.66	1.60	0.51	1.49
	Recall	0.33	0.86	1.00	0.27
	Precision	0.36	0.83	0.95	0.18
	F1 score	0.35	0.85	0.98	0.21
Length metric	Recall	0.00	0.47	1.00	0.00
	Precision	0.00	0.50	0.54	0.00
	F1 score	0.00	0.48	0.70	0.00

**Figure 4:**
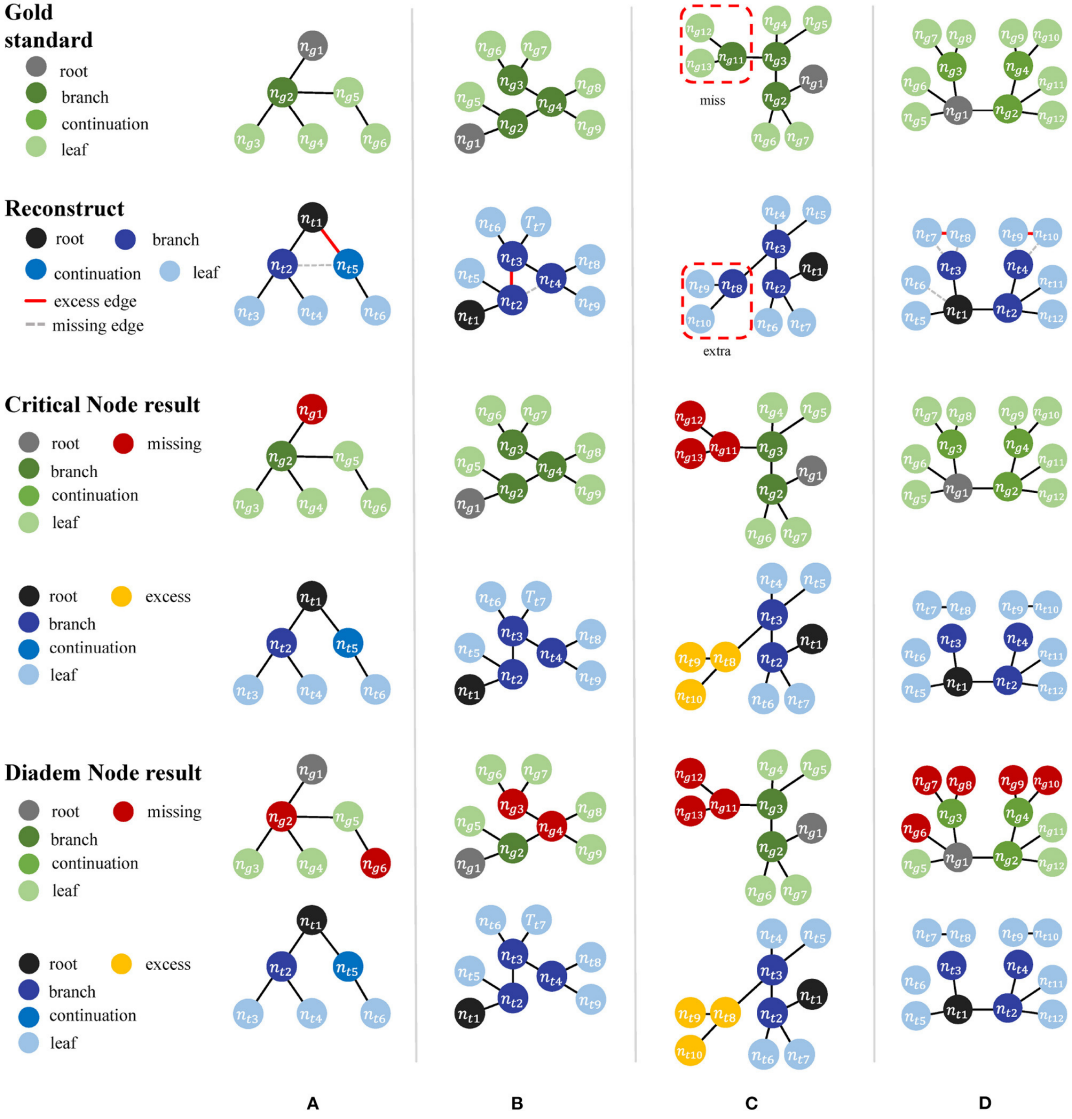
**(A–D)** Four manually constructed cases for testing topological metrics.

The difference between the two topological metrics can be seen in Figure 4 and Table 4. The CN metric fails to detect reconstruction errors in Figures 4A,D because the errors do not add or remove a critical node. The DIADEM metric can avoid such a problem by including path comparison. In this sense, the DIADEM metric is more comprehensive than the other three metrics in PyNeval as it actually considers both topological and geometrical features. Nevertheless, we should note that it may not correlate well with the amount of editing work needed to fix errors. For example, the test model in case 2 can be more readily fixed than case 3, despite that it has a lower DIADEM score. In other words, the DIADEM metric can be misleading when we expect an automatic method to minimize manual work.

**Table 4:** PyNeval results of the DIADEM and length metrics for the topological cases are shown in Figures 4A–D.

**Method**	**Index**	**file name**			
		**A**	**B**	**C**	**D**
Diadem metric	Score	0.625	0.56	0.69	0.72
Critical node metric	Recall	0.80	1.00	0.70	1.00
	Precision	1.00	1.00	0.70	1.00
	F1 score	0.89	1.00	0.70	1.00

### 3.3. Reconstruction Parameter Optimization Using PyNeval

Besides comparing different reconstruction algorithms, another important application of PyNeval is to optimize parameters of the same tracing algorithm. We can treat this as a numerical optimization problem. For any tunable reconstruction program 𝔓(*I*|**θ**), in which image *I* and parameteres **θ** are inputs and SWC model is the output, we define the optimization problem as


(20) $$\begin{array}{l} \underset{textbf\theta }{\min}\text{E}\left(L\left(𝔓\left(I|\theta \right),T_{g}\left(I\right)\right)|I\right) \end{array}$$

where L is the loss function that can be computed from reconstruction metrics.

**Figure 5:**
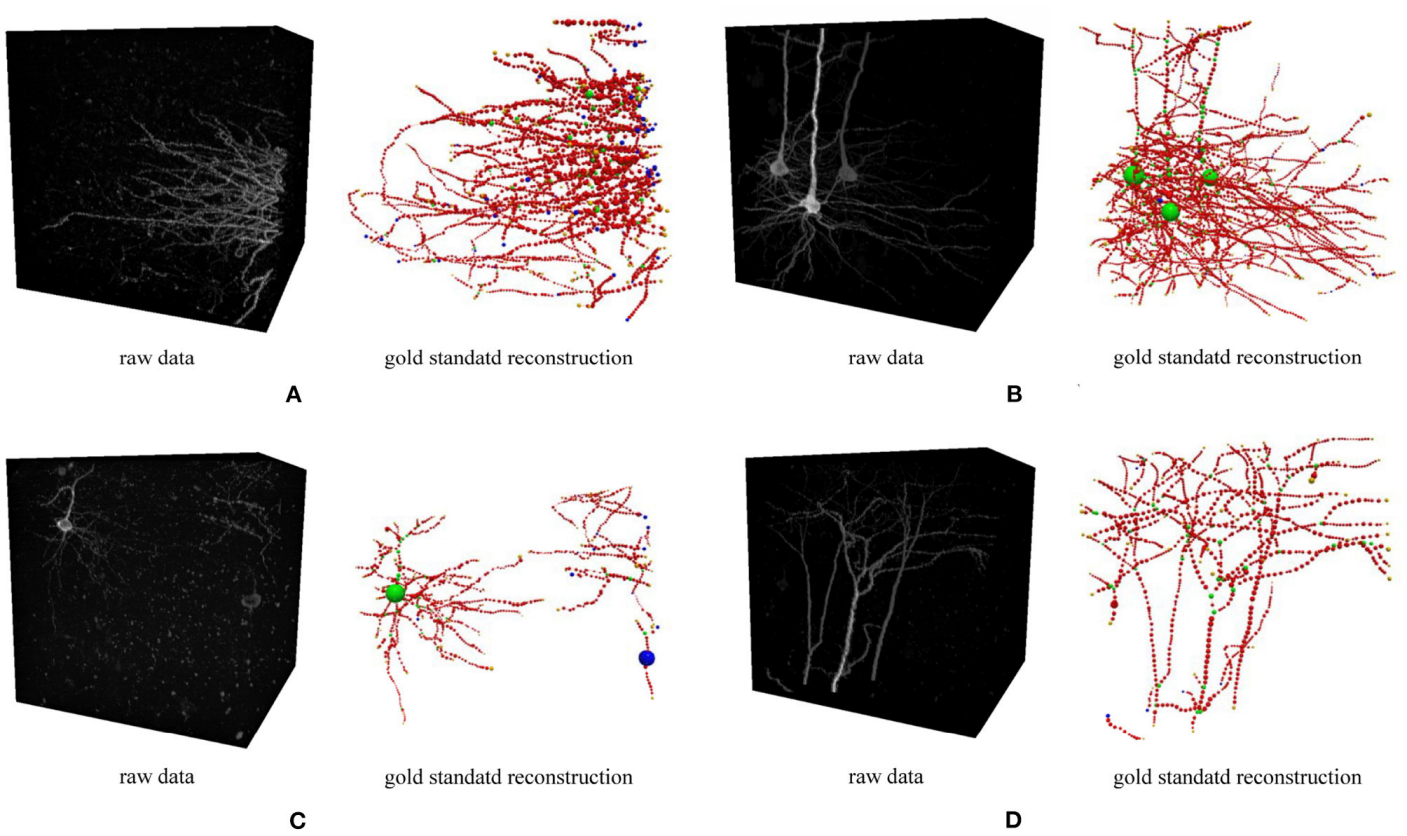
Four 3D neuron images used in the optimization experiment. Each image and its gold standard reconstruction is rendered side by side in each panel labeled by the corresponding dataset ID. **(A)** FM3, **(B)** FM4, **(C)** FM5, and **(D)** FM6.

In real applications, we expect parameters optimized on a training dataset can be generalized to other images from the same imaging protocol. Therefore, we carried out a cross-validation experiment on four image blocks (Figure 5) from a whole mouse brain sample acquired by fMOST (Gong et al., 2016). The cross-validation searched for the best parameters for each block and used these optimized parameters to trace other blocks. In our experiment, we used the F1 score of the SSD metric as the loss function to optimize the automatic neuron tracing method used in neuTube (Zhao et al., 2011), which has two numerical parameters for adjusting the sensitivity of branch detection. The optimization process was performed by simulated annealing (Van Laarhoven and Aarts, 1987), which searches the parameters iteratively. A new parameter θ^(*k*+1)^ at the *k*th iteration was calculated by


(21) $$\begin{array}{l} \theta^{\left(k+1\right)}=\theta^{\left(k\right)}+20\frac{u}{\left|u\right|}*t_{k}*\left({\left(1+\frac{1}{t_{k}}\right)}^{\left|u\right|}-1\right) \end{array}$$

where *u* was drawn randomly from [−1, 1]\0 and *t*_*k*_ was the temperature at the *k*th iteration. Starting from *t*_1_ = 0.01, the temperature was decreased every 25 iterations at the rate of 0.96. The stop criterion was that the temperature was below 10^−5^ or the optimal value had not been improved for 20 iterations.

**Figure 6:**
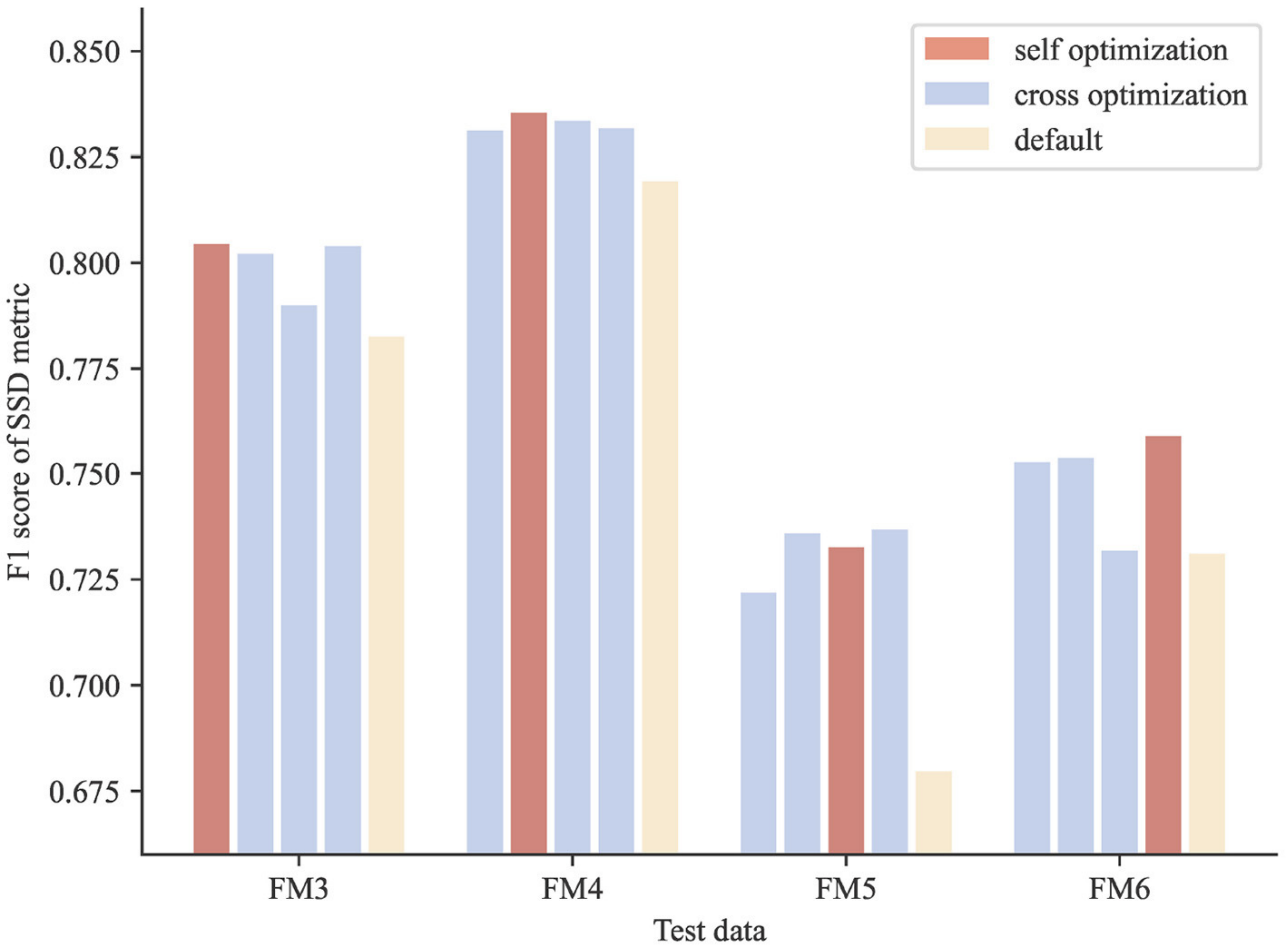
Cross-validation results of parameter optimization for neuron reconstruction. The scores of the optimized parameters are consistently better than those of the default parameters for all the test images.

The results show that the optimized parameters outperformed the default parameters consistently, no matter which image block was used in parameter searching (Figure 6), presenting a successful example of using PyNeval in improving automatic neuron tracing.

## 4. Conclusion and Future Work

Motivated by the difficulties of evaluating automatic neuron tracing methods, we have developed PyNeval, a user-friendly Python toolbox to help method developers focus on algorithm development and method users choose a proper method for their own applications. PyNeval has made four popular metrics that cover both the geometrical and topological categories easily accessible to the community. A user can easily install PyNeval through common Python package managers and run the program as a command line with a straightforward but flexible interface. We have also shared the source code of PyNeval on https://github.com/CSDLLab/PyNeval to show how the metrics were implemented exactly as well as inspire further development.

To facilitate further development, PyNeval has a well-modularized architecture for maximizing its extensibility. It is straightforward to add more metrics such as the NetMets metric (Mayerich et al., 2012) in the future while keeping backward compatibility. Another important plan for further development is to make PyNeval an easy-to-use Python library as well, so that, other users can easily call functions in PyNeval from Python code directly, or even contribute their own metrics to PyNeval.

## Data Availability Statement

The raw data supporting the conclusions of this article will be made available by the authors, without undue reservation.

## Ethics Statement

The animal study was reviewed and approved by Zhejiang University.

## Author Contributions

TZ and NZ designed and supervised the project. HZ wrote most part of the software with help from YY and TZ. HZ, CL, and JD performed data analysis. HZ, TZ, and NZ wrote the manuscript. All authors contributed to the article and approved the submitted version.

## Funding

This work is supported by the National Key R&D Program of China (2020YFB1313501), Zhejiang Provincial Natural Science Foundation (LR19F020005), National Natural Science Foundation of China (61972347, 61976089), and Hunan Provincial Science & Technology Project Foundation (2018RS3065, 2018TP1018).

## Conflict of Interest

The authors declare that the research was conducted in the absence of any commercial or financial relationships that could be construed as a potential conflict of interest.

## Publisher's Note

All claims expressed in this article are solely those of the authors and do not necessarily represent those of their affiliated organizations, or those of the publisher, the editors and the reviewers. Any product that may be evaluated in this article, or claim that may be made by its manufacturer, is not guaranteed or endorsed by the publisher.
